# Prevalence and Risk Factors of *Toxoplasma gondii* in Fattening Pigs Farm from Yucatan, Mexico

**DOI:** 10.1155/2013/231497

**Published:** 2013-06-06

**Authors:** A. Ortega-Pacheco, K. Y. Acosta Viana, E. Guzmán-Marín, J. C. Segura-Correa, M. Álvarez-Fleites, M. Jiménez-Coello

**Affiliations:** ^1^Universidad Autónoma de Yucatán, FMVZ, Campus de Ciencias Biológicas y Agropecuarias, Carretera Mérida-Xmatkuil Km. 15.5, Apdo. Postal 4-116 Itzimná, 97100 Mérida, YUC, Mexico; ^2^Universidad Autónoma de Yucatán, Centro de Investigaciones Regionales “Dr. Hideyo Noguchi” Unidad Biomédica, CA. Biomedicina de Enfermedades Infecciosas y Parasitarias, Laboratorio de Biología Celular, Avenida Itzáes No. 490 x Calle 59 Colonia Centro, 97000 Mérida, YUC, Mexico

## Abstract

The objective of this study was to estimate the prevalence and identify risk factors associated with the presence of *Toxoplasma gondii* in pig-fattening farms from Yucatan, Mexico. A cross-sectional study was conducted with a two-stage sampling. There were 429 pigs sampled from 39 farms randomly selected. Blood samples were collected to obtain DNA and serum. The presence of IgM and IgG antibodies was determined by indirect ELISA. Prevalence was estimated by diagnostic test. Potential risk factors to be included in a marginal logistic regression were tested by chi-square or Fisher. The prevalence of IgM and IgG was 92.5% (397/429) (CI 89.9–95.1%) and 95.8% (411/429) (CI 93.7–97.8%), respectively. Regarding PCR, a prevalence of 50.8% (218/429) (CI 45.9–55.6%) was found. The logistic regression showed an association with herd size and type of feeder (*P* < 0.05). The risk of a case in farms with ≤400 pigs was 27.9 times higher than in farms with >400 pigs. The manual feeder was a significant protective factor associated with the seropositive against *T. gondii*. Results indicate a high circulation of *T. gondii* in pig-fattening farms from Yucatan, finding an increased risk of infection for those farms with less than 400 animals and automatic feeders.

## 1. Introduction

Toxoplasmosis is an infectious disease caused by the protozoan parasite *Toxoplasma gondii* (*T. gondii*), being the pig among other animals intermediate hosts [1]. This is a zoonotic disease with a high impact on public health [2]. Human infections may go unnoticed or may cause various signs and symptoms depending on the patient's immune status and general health status (i.e., immunocompetent state, eye disease, congenital toxoplasmosis) [3]. Reactivation of disease and infection to the central nervous system (CNS) occurs in immunosuppressed patients resulting in severe encephalitis [4]. The toxoplasmic chorioretinitis in humans can be congenitally or postnatally acquired as a result of an acute infection or reactivation of the disease [5]. Congenitally infected fetuses with toxoplasmosis may develop hydrocephalus, microcephaly, intracranial calcifications, chorioretinitis, strabismus, blindness, epilepsy, mental retardation, and anemia among others [6]. 

 The route of infection with *T. gondii* in man and animals is by incidental ingestion of oocysts from the feces of cats. Oocysts are highly resistant to environmental conditions and contaminate water, soil, dust, vegetables, and fruits [7]. However, infection through the ingestion of tissue cysts in meat is considered one of the main sources of infection to humans. Between 30% and 60% of pregnant women who consumed inadequately cooked meat may suffer from acute toxoplasmosis [8]. The low prevalence of toxoplasmosis found in a group of vegetarians (24%) confirms the suspicion that consumption of meat is one of the most important ways of transmission of *T. gondii* to man [9].

Swine plays an important role in the transmission of infection to humans [10]. A study reveals that, in the period 1983-1984 in the United States, 23.9% of pigs presented specific titers against *T. gondii;* from those, 42% were breeders and 23% were commercial fattening farms [11]. By 1992, prevalence in the same region dropped to 20.8% in breeders and 3.1% in fattening pigs due to changes in their production systems and preventive measures taken [12]. Toxoplasmosis is found in different animal production systems in Mexico. In pigs sampled in central Mexico, 8.9% were positive [13]. The mortality associated with toxoplasmosis in pigs is greater in young than in adult pigs. It is also responsible for pneumonia, myocarditis, encephalitis, and placental necrosis in this species [14]. 

Seroepidemiological studies have demonstrated the huge impact of pork contaminated with tissue cysts on the transmission to humans from this disease [15, 16]. Also, it is known that a single pig intended for consumption that is contaminated with cysts in muscle tissue is capable of transmitting infection between 200 and 400 individuals [17]. On the other hand, molecular studies may demonstrate the presence of circulating genome from the parasite given a broader panorama of the epidemiological situation of *T. gondii* in the studied population.

The State of Yucatan is an important pork producer in Mexico, with about 95.933 tons of pork each year, and has a per capita consumption of 10 kilos per year [18]. However, there is little information on the presence of *T. gondii* in pig farms and even more in pork intended for consumption. Therefore it is necessary to conduct epidemiological studies to determine the situation in pig populations in the region, with the aim of establishing prevention and control measures to reduce their impact at farm level and public health risk. 

The aim of this study was to estimate the prevalence and risk factors associated with the presence of* T. gondii *in pig-fattening farms in the state of Yucatan, Mexico, destined for human consumption, by serological and molecular detection of the etiological agent.

## 2. Material and Methods

### 2.1. Study Area

The study was conducted in the state of Yucatan, located in southeastern Mexico (19° 30′ and 21° 35′ north latitude and 90° 24′ west of the meridian of Greenwich). The climate is tropical subhumid with summer rains. The maximum monthly temperature varies between 35 and 40°C, with an average temperature of 26.6°C. The relative humidity varies between 65 to 100% taking the mean value over 78%. The annual rainfall is from 415 to 1290 mm [19].

### 2.2. Sample Collection

A two-stage cross sectional study was performed during September to December 2008. Four hundred and twenty-nine pigs from 39 farms, between 18 and 20 weeks of age, were randomly selected. The sample size to estimate prevalence was determined by the formula: *n* = *D*(*z* ^2^ *p*(1 − *p*)/*d* ^2^), considering a confidence level of 95%  (*z* = 1.96), absolute precision of 5%  (*d*), a design effect (*D*) of 2, and a prevalence of 25%  (*p*). The estimated prevalence was obtained from a pilot study that included 45 animals. The number of animals sampled at each farm (*b* = 11) was calculated based on the formula *b* = √(ce/cd)(1 − re/re), where ce is cost of a sample into two clusters (10); cd, sample cost of two units of interest in a same cluster (1) and the correlation intra-conglomerates (re) was (0.04) [20]. The number of farms (*m* = *n*/*b*) was calculated by dividing the sample size (*n* = 429) between the numbers of animals sampled in each establishment (*b* = 11).

### 2.3. Serum and DNA Extraction from Blood

Two blood samples per animal were collected in vacutainer tubes with and without EDTA for subsequent DNA extraction and to obtain serum, respectively. Samples for serum collection were centrifuged at 2500 g for 10 min and were stored at −20°C until further evaluation.

Genomic DNA was extracted using the QIAamp DNA mini kit (Qiagen, CA, USA). Before extraction, a pre-lysis of the sample was conducted as suggested by Jalal et al. [21]. Samples were stored at −20°C until further PCR assay.

In order to verify the viability of purified DNA samples, PCR was performed establishing the *β* actin gene. Amplification conditions were 20 pM of each primer: *β*1 5′-ATCTTGATCTTCATGGTGCTGGGC 3′ and *β*2 5′-ACCACTGGCATTGTCATGGACTCT3′ [22], containing 1 U of enzyme GoTaq Hot Start Polymerase (Promega, WI, USA), 1X PCR Buffer Colorless GoTaq Flexi Buffer, and MgCl_2_ at a concentration of 2 mM in a final reaction volume of 25 *μ*L. The alignment temperature was 60°C, awaiting a product size of 545 bp [22].

### 2.4. Serology

The presence of specific IgM and IgG antibodies against *T. gondii* was determined separately by the use of indirect ELISA tests (Human-GmbH, Wiesbaden, Germany), on a 96-well plate coated with tachyzoites of *T. gondii*. Serum samples were diluted to a ratio of 1 : 100 in phosphate-buffered saline (PBS; pH 7.2). A secondary goat anti-IgG pig antibody labeled with peroxidase (HRP) (Santa Cruz Inc. CA, USA) and a goat anti-IgM pig also marked with HRP (Serotec, Oxford, UK) were used, respectively, at a dilution of 1 : 5,000. Sera from pigs showing high anti-IgG antibodies titer by ELISA (1 : 1024) and positive results to PCR against *T. gondii* were used as positive controls, and sera pools from 10 healthy pigs previously tested by triplicate with ELISA IgM, IgG, and PCR were used as negative controls. On the basis of the ELISA, subjects were diagnosed as either positive/negative for specific IgG and IgM antibodies to *T. gondii*. The optical density (OD) was measured in a spectrophotometer at 450 nm (Multiskan Multisoft Primary EIA) and was used to compute the percent positivity (PP) using the formula mean OD (sample or negative control) divided by the mean OD value positive control multiplied by 100. Percent positivity of 15% or above was considered positive. 

### 2.5. Polymerase Chain Reaction for *T. gondii* Detection

Conventional PCR was performed to amplify the B1 gene of *T. gondii*, which is highly conserved in the parasite. Primers Tg1 (5′-AAAAATGTGGGAATGAAAGAG-3′) and Tg2 (5′-ACGAATCAACGGAACTGTAAT-3′) were used because of the high specificity (100%) for the B1 gene [21] of *T. gondii*. Amplification conditions were 40 pM of each primer, 1 U of GoTaq enzyme Hot Start Polymerase (Promega, WI, USA), containing 1X PCR Buffer Colorless GoTaq Flexi Buffer and MgCl_2_ at a concentration of 0.8 mM, in a total volume of 25 *μ*L per reaction. The alignment temperature was 51°C and an amplified product of 469 bp was obtained. The PCR protocol was performed as follows: denaturation at 95°C for 10 min, followed by 35 cycles of 95°C, 52°C, and 72°C during 60 sec, 30 sec, and 60 sec, respectively—with a final elongation at a temperature of 72°C during 7 min. For the PCR performance, a thermal cycler was used (Applied Biosystems, Foster City, USA). Subsequently the PCR products were analyzed by electrophoresis on agarose gels and stained with ethidium bromide 1.8% (10 mg/mL) for 15 minutes and visualized with UV light, imaged them using a photo document (Applied Biosystems, Foster City, USA). As a positive-control PCR assay, the amplification of a clone obtained in the laboratory was carried out (PmosBlue plasmid with a PCR amplificate as an insert of the B1 gene of *T. gondii* RH strain). The bacterial clone was transformed into *E. coli* strain TOP10 and was purified with a commercial kit (Roche High Pure Kit, Manhein, GER). From the elution obtained, 32 ng × 10^6^ of the clone was used as a positive control in the PCR reaction.

### 2.6. Risk Factors

The information on risk factors was obtained by applying a questionnaire. The risk factors considered were herd size (≤400 and >400 pigs), presence or absence of cats, cats number (≤3 cats, >3 cats, and no presence of cats), presence of rodents (yes/no), pest control (yes/no), type of feeder (automatic/manual), cannibalism (yes/no), production system (complete cycle/fattening), and place of storage food (warehouse/silo).

### 2.7. Statistical Analysis

The adjusted prevalence by herd size was estimated by the formula *p* = Σ*N*_*i*_ *p*_*i*_/*N*, where *N*_*i*_ is the size of the *i*th farm, *p*_*i*_ is the prevalence of the *i*th farm, and *N* is the total number of pigs in the studied population. Confidence interval (95%) was also calculated [20].

A positive pig was defined as one positive to IgG ELISA or PCR. Contingency tables were constructed to identify those risk factors with cells with zeros, which were discarded for further analysis. Factors that were significant (*P* < 0.20) in the Chi-square or Fisher tests were included in a binomial logistic regression model adjusted for the effect of farm using the procedure GENMOD [23].

## 3. Results

### 3.1. Serology

The seroprevalence of IgM anti-*T. gondii* found was 92.5% (397/429) (CI 89.9–95.1). From the sampled farms, 36 showed seroprevalence of 100% and 3 of 90%. Regarding IgG antibodies, a prevalence of 95.8% (411/429) (CI 93.7–97.8) was found; 33 farms showed prevalence of 100%, 1 farm of 90%, 3 farms with prevalence between 30 and 70%, 1 farm with prevalence <30%, and 1 farm seronegative to *T. gondii.*

### 3.2. PCR for *T. gondii* Detection

**Figure 1:**
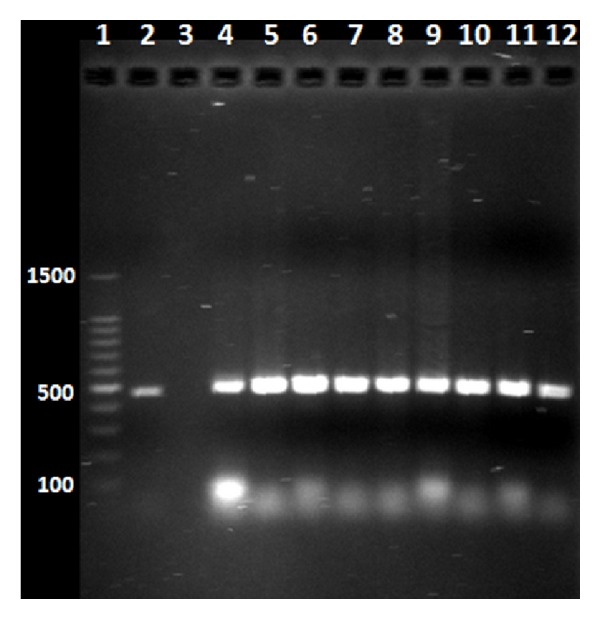
Agarose electrophoresis (1.8%) with BrEt staining showing the PCR amplified products of B1 gene from *Toxoplasma gondii*. Lane 1: molecular weight marker (100 bp. Promega), lane 2: positive control of *Toxoplasma gondii* RH strain, lane 3: negative control (uninfected pig), lanes 4–12: positive pigs from Yucatan, Mexico.

A *T. gondii *prevalence of 50.8% (218/429) (CI 45.9–55.6) was found with the PCR technique. Of the 39 farms, nine had a prevalence of 100%, five were positive in 90%, seven showed values between 50 and 90%, and 18 farms' prevalence found was between <50 and 0%. An example of positive pigs identified by PCR and electrophoresis is shown in Figure 1.

Two hundred and eleven pigs were positive to ELISA (IgM and IgG) as well as in PCR, showing a total prevalence of *T. gondii* with these 3 tests of 49.1% (95% CI 45.9–55.6) (Table 1). The amplified products were purified and were sequenced, showing homology of >99% identity with the B1 gene of *T. gondii*.

### 3.3. Risk Factors

The risk factors farm size, food storage, and type of feeder showed statistic association by univariated analysis (*χ* ^2^). Presence of cats, cats number, cannibalism, and production system the showed significant values (*P* < 0.05) but were not considered in the logistic regression model because of the low number of cases in contingency tables. Also, the variable presence of rodents was significant but it was not included in the multivariate study because the data obtained in the contingency table were not consistent (Table 2).

The logistic regression model adjusted for farm showed association with herd size and type of feeder (*P* < 0.05). The risk of a case on farms with ≤400 pigs was 27.9 times higher than on farms with >400 pigs. The manual feeder was a significant protective factor associated with the seropositive towards *T. gondii* (Table 3).

## 4. Discussion

Results obtained in this study showed a high circulation of *T. gondii* in fattening pigs on farms in the state of Yucatan, Mexico. The prevalence of IgG antibodies (95.8%) is higher than the 8.9% found by García-Vazquez et al. [13] on commercial pig-fattening farms from central states of Mexico. Toxoplasmosis in pigs has a wide worldwide variation, ranging from 2.7% in the USA [24] to 37% in Brazil [25], and can show a yearly variation of 11.6% in 2001, 0% in 2003, and 1.2% in 2004 as reported in Canada [26]. These wide variations occur as result of different risk factors and control measures adopted in the pig production systems from each country.

There are few epidemiological studies in naturally infected pigs using immunoglobulin IgM. The study of IgM and IgG immunoglobulins at the same time in the study population may demonstrate the dynamic response of these antibodies during natural infections with *T. gondii*. During experimental infections in pigs, IgM reached a peak 10 days after infection and remained 21 to 24 days later. The persistence of IgM after the acute phase of infection may be normal for this immunoglobulin [27]. The IgM immunoglobulin was found in most of the pigs sampled even in the presence of IgG. This finding suggests that pigs were probably in a state of reinfection or persistence of antigenic stimulation of the agent. 

It is known that IgM immunoglobulin is produced during the acute phase of toxoplasmosis [28] and can also be observed during secondary responses (chronic phase in the presence of IgG), only being hidden by the predominance of the latter [27, 29]. The possibility of reinfection of pigs may explain the PCR results found since this technique directly identified the presence of the genome of the parasite in the blood samples [30]. Serological tests only explain previous contact with the agent. When placed together, the molecular and serological status may indicate the phase of infection. In cats, the presence of IgM or PCR-positive *T. gondii* cases may indicate acute cases, whereas IgM + IgG + PCR positive cases indicate chronic reactivated cases [31]. In the present study almost half of the studied animals were in the chronic reactivated stage indicating a constant exposure.

The likely source of reinfection of pigs can be caused by constant contact with infective oocysts of *T. gondii* present in the farms, either in water sources, soil, or air, which is commonly found in swine production systems [32]. *T. gondii* oocysts can be viable for a couple of years (540 days) and tolerate extremes of temperature and humidity promoting their persistence in the environment [33], and they are capable of producing infection by contact with susceptible animals. Likewise, the presence of cats in farm production systems can increase the spread of pollutant oocysts. Moreover, the presence of agents causing immunosuppression in farms such as arterivirus (PRRS), *Mycoplasma hyopneumoniae* (enzootic pneumonia), and *Circovirus* (PCV2) [34, 35] could cause the reactivation of cysts tissue [36]. A case of systemic toxoplasmosis associated with concurrent infection with *Circovirus* in a pig fattening farm has been reported, probably due to immunosuppression caused by the virus [37]. 

A higher risk of infection by *T. gondii* in those farms where the population size was less than 400 individuals was identified, similarly as reported by Assadi-Rad et al. [38] in farms with a population of less than 29 breeding females. Also Villari et al. [39] found that the prevalence in pig farms decreased beyond 50 animals. This could indicate that small farms increased risk of exposure to the agent as risk factors are distributed in few animals [40].

So it is likely that in the most densely populated farms, the hygiene, intensive management and more infrastructures can lead to the reduction of *T. gondii* in the environment [41]. 

Although in this study the presence of cats was not significantly associated with *T. gondii*, prevalence results suggest that cats may have an important role, and although they were not currently present, oocysts contamination may persist in the farms; feline species is crucial to increasing environmental pollution [26, 39, 42]. It is important to consider that other risk factors such as cannibalism have proved to be another route of infection of *T. gondii* when pigs eat tissue cysts from rodents or from other pigs. Place of food storage is another factor to take into consideration. In outdoors or in warehouses without control of cats, food contamination by oocysts may happen [10, 12, 39, 43].

In the present study, manual feeder was protective against the presence of *T. gondii* probably because in automatic feeders, the food is maintained for longer periods, increasing the possible contamination with oocysts or oocysts eliminated by cats; surplus of food in manual feeders is more regularly cleaned for the replacement of a new ration.

The findings reported here have important public health implications as they suggest that the pork produced in the Yucatan for human consumption is an important source of contamination with great potential for transmission of *T. gondii*. It is important to consider that children, pregnant women without any previous contact with the agent, immunosuppressed people, and individuals working in pig farms and slaughterhouses are vulnerable groups where prevention is essential [40, 44]. 

We conclude that there is a high presence of *T. gondii* in intensive pig-fattening systems of the state of Yucatan. There are risk factors that promote their presence causing reinfection and factors that limit their contact in pigs. The level of infection must be reduced on pig farms with special emphasis on the control of cats and rodents.

## Figures and Tables

**Table 1:** Serological and molecular status of fattening pigs in Yucatan, Mexico.

	IgM (−)	IgM (−)	IgM (+)	IgM (+)	IgM (+)	IgM (+)	
	IgG (−)	IgG (+)	IgG (+)	IgG (+)	IgG (−)	IgG (−)	*n*
	PCR (−)	PCR (−)	PCR (−)	PCR (+)	PCR (+)	PCR (−)	

	3	29	171	211	7	8	429

*p* (%)	0.7	6.75	39.8	49.1	1.63	1.86	100%

*p*: prevalence.

**Table 2:** Variables measured in the study of cross section for *T. gondii* in fattening pigs from 39 farms in the state of Yucatan, Mexico.

Variable	Category	Positive	Negative	Chi^2^ value	*P*
Farm size	≤400	272	3	45	0.0001
	>400	125	29		
Presence of cats	Yes	301	29	4	0.055
	No	96	3		
Cats number	>3	176	22	7	0.026
	<3	136	7		
	0	85	3		
Food storage	Ensilage	31	13	34	0.001
	Warehouse	366	19		
Type of feeder	Automatic	75	13	9	0.003
	Manual	322	19		
Cannibalism	Yes	155	0	19	0.0001
	No	242	32		
Production system	Full cycle	309	32	9	0.0028
	Fattening	88	0		
Presence of rodents	Yes	95	147	28	0.000
	No	122	65		
Pest control	Yes	109	108	0.19	0.65
	No	111	101		

**Table 3:** Logistic regression analysis adjusted for the effect of farm for *T. gondii* in 429 fattened pigs from 39 pig farms in the state of Yucatan, Mexico.

Risk factor	b	EE	OR	95% IC	*P*
Size of the farm					
≤400	3.33	0.76	27.9	6.29–125.2	0.0001
>400	0		1		
Food storage					
Ensilage	0		1		
Warehouse	−0.23	0.71	0.11	0.19–3.18	0.74
Type of feeder					
Automatic	0		1		
Manual	−1.67	0.70	0.18	0.04–1.32	0.018
